# Remarks on the Diseases of Ohio, in a Letter to the Editors

**Published:** 1845-12

**Authors:** John Dawson

**Affiliations:** Jamestown, Ohio


### WESTERN JOURNAL

OF

### MEDICINE AND SURGERY.

---

DECEMBER, 1845.

---

### Art. I.—*Remarks on the Diseases of Ohio, in a letter to the Editors.* By John Dawson, M.D., of Jamestown, Ohio.

Gentlemen:—Supposing that you would like to know something of the diseases with which the interior of Ohio is visited at the present time, and having no leisure to embody my remarks in the form of an essay, I will give a mere epistolary sketch of some of the principal ones that have prevailed.

From the mouth of every body the report comes, that this is a *sickly season*, more so, in this section, than has been experienced for a number of years. Without doubt, I am inclined to think, that the report is correct. We have certainly more disease at present, than has been witnessed for a long time, perhaps ten years; and, indeed, some think more than has been, in any one year, since the country was settled.

As the diseases are chiefly of miasmatic origin, I will glance briefly at some of the circumstances connected with their prevalence in our district.

You are aware that we are situated on the table-lands dividing the waters of the Little Miami and Scioto rivers, and therefore pretty remote from anything like extensive alluvial deposits. In this region, the country is, however, level, and the streams running to each of the rivers are so shallow, that they have been inadequate to conduct off the water proceeding from the heavy rains with which we have recently been visited. The circumstances therefore have been what are generally considered favorable to the generation of malaria.

The past winter and spring were unusually dry, with a temperature decidedly mild. So great was the drought in May and June, that an entire failure of crops was anticipated. The latter part of the month of June, and all of July were attended with more seasonable weather, such as dissipated the fears of the farmer, and gave promise of a sufficient supply of all the necessaries of life. Since the seasonable weather commenced, we have been visited regularly with copious rains, and that too at short intervals. These have kept the vegetative process so very active through the months of August and September that almost every species of plant has attained a luxurious growth.

As the growth of plants was rapid, the work of decomposition commenced going on in the latter part of July, and as a consequence we have had more than the usual quantity of disease ever since. Indeed it has gradually increased until the number afflicted is said to exceed that of any one year since the country was inhabited. Among those diseases which have assumed the most importance, is

BILIOUS FEVER.

There has been very little of this disease in this region for the last four or five years. Old practitioners say, much less than formerly. I saw a few cases of it in July, more in August, and in the month of September it seemed to attain its greatest prevalence. The *pyrexia*, as a general rule, has been decidedly inflammatory, the circulation being rapid, and in most cases the pulse very full; the skin hot and dry, and often very yellow. The alimentary canal was very susceptible to purgatives, though it exhibited no tendency to take on irritation and run into gastro-enteritis; nor was there much trouble from irritation in the stomach, giving rise to bilious vomitings. Disordered *liver* was prominent in every case, showing itself in bilious discharges from the bowels, a yellowness of the skin and eyes, and a brownish color of the coat on the tongue. The *duration* of most cases has been short. Three or four days has been sufficient, in a majority of instances, to bring the disease to a close. A few cases went into the typhoid stage, and lasted some fifteen or twenty days. These, however, were rare, and I think were due oftener to a want of medicines at the proper time, than to any peculiarity of the complaint, or of the remote cause.

Of the *complications* of this disease with the epidemic, (erysipelas) with which it was preceded in our neighborhood, I witnessed but little. Three cases that I saw were complicated with some of the phenomena of epidemic erysipelas. They yielded, however, as readily to remedies as any that I witnessed.

Into a lengthy consideration of the *treatment* it is unnecessary for me to enter. Bloodletting was premised in all cases where it seemed to be justifiable, as for instance in plethoric individuals, or where the action of the heart and arteries was such as to interfere with any of the great functions of life. When such was not the case, the treatment was commenced with emetics, (ipecac. and tartar emetic) or purgatives. As soon as the first passages were cleared, without waiting for any prominent remission in the symptoms, I gave quinine in five or six grain doses, in combination with carbonate of iron. This combination was generally administered in the morning, about two doses of which were taken every day until the fever subsided. The bowels were kept open with Seidlitz powder, neutral salts, or jalap.

Concerning the results of this practice, for it is the first time I ever adopted it, I may state—1. That the fever was of shorter duration than when treated by the ordinary method of elementary writers. 2. That it left the system more free from the *sequelæ* of either disease or treatment, and as a consequence the convalescence was more rapid; and what is of most importance, it was entirely successful, so far as tried.

The plan of treating bilious fever with mercurial cathartics and ptyalism, originated, likely, in the supposition that cholegogues to restore the functions of the liver would do more to check the disease, than anything exercising a more direct antipyretic effect. This motive, plausible as it may seem from the prominent share which the liver takes in the pathology of bilious fever, is about to be changed. The preparation of Peruvian bark, whether considered as an antiperiodic, or an antipyretic, contains something that neutralizes or serves as antidote to the poison of our autumnal fevers. This fact may be considered as well settled, not only in regard to intermittent and bilious remittent fever, but also in regard to yellow fever.

For experience to determine, a matter is still left. I allude to the *time*, during the course of the bilious fever, at which quinine should be administered. With all, I agree, that when a remission can be obtained, that is the most auspicious. When, however, there is nothing like a decided remission, which is very unusual, the medicine should still be given. The mornings should, I believe, be selected, as, *cæteris paribus*, the fever will be found less at that time, than at any other. Given even during the exacerbation less disturbance will follow than has been generally anticipated. At all stages of the fever I have administered it with impunity. When I can get a remission, of course, I prefer it. But I think we are not justifiable in waiting for it when the symptoms are urgent.

### INTERMITTENT FEVER.

This disease made its appearance early in the summer, and I think attained its greatest prevalence in September. Usually it was of the tertian type. I witnessed, nevertheless, a number of cases of the quotidian, and a few of the *double* tertian. As a general remark the disease was not malignant. A few cases in our county assumed the congestive form, common in hot climates, and well described by Alibert. These, as far as I have learned, generally went to a fatal issue in two or three paroxysms. I attribute this result to the fact that this form of intermittent fever is of very unusual occurrence here, and as a consequence, neither the people nor the physicians are aware of its danger until it is often too late. I saw a case some years since in which the patient, for a week or two, had suffered from what he regarded as being an ordinary ague. At a certain time when he was expecting his chill, it came on followed by great congestion of the head and deep-seated organs of the body. He became delirious, with small pulse, cool extremities, laborious respiration, and slight heat of skin. In this situation he continued for about seventy-two hours and died. This was an ordinary case that degenerated into a malignant intermittent. Some, however, have occurred that were of the malignant character from the commencement.

Of intermittent fever complicated with erysipelas, I have seen but one case. For a while, this presented an array of very formidable symptoms, but under the use of medicines, addressed principally to the fever, it yielded.

Concealed intermittents, or what are sometimes termed "*masked agues*" have prevailed this season, under the form of periodic neuralgia of the face, side of the head, forehead, extremities, &c. These anomalous affections, I have no doubt, owed their origin to the same cause which produced the miasmatic fevers, for the means of cure were identical.

Into bilious continued fever, Armstrong says he has seen intermittents frequently run. This is a result, that I have looked for with some degree of attention since these fevers have been prevailing, but as yet I have not noticed it to take place. The contrary, the termination of bilious continued fever in intermittent fever, has been a result, the present season, of frequent occurrence.

To the cause of these autumnal fevers inquiry has frequently been directed; but so far, such labor has been fruitless. One fact, however, in regard to this matter may now be regarded as settled. I allude to the identity of cause producing bilious continued and intermittent fever. There can be no doubt, whatever it may be, that it is one and the same thing which produces both fevers. This fact is of great importance in the treatment of bilious fever. Heretofore attention has been almost exclusively directed to the circulation, to the state of the secretions, and to the alimentary canal. Bark was scarcely thought of, unless the fever was first converted into an intermittent. Now we look upon the preparations of this article as being as salutary in the continued, as in the intermittent form of fever, and its introduction into practice, as being the greatest modern improvement, (the *feeding* plan of managing adynamic fevers, excepted) that has taken place in the treatment of fever.

A circumstance connected with the prevalence of these autumnal fevers, is that a region of country may be almost completely exempt from them for a number of years, when without any apparent cause or change of circumstances they may make their appearance again in almost the character of an epidemic. This region in 1839,–'40,–'41,–'42,–'43, suffered but little from bilious diseases of any kind, and particularly bilious fever. In 1844 we had none of them; and during the present year they have attained a prevalence hitherto unknown.

Intermittents are treated almost exclusively in this region with quinine. Some years since, I gave sulphate of zinc a trial, but in its effects I was disappointed. If it possesses any power to break up the laws which keep an intermittent revolving in the system, this is but weak in doses that are admissible. I have given it frequently in combination with quinine to see if it would not be more powerful in preventing relapses. I can report nothing here in its favor. The black spider's web, so highly spoken of by Dr. Robert Jackson, I have tried in a number of cases without witnessing any benefit from its use. McIntosh's plan of bleeding in the cold stage has been practised here, and with very good results. When intermittents are congestive it is first rate practice, not only scientific but really successful. The administration, however, of quinine during the apyrexia, should not be neglected.

The treatment of the malignant intermittents which have appeared here, has differed in no very essential respect from that adopted in the mild form of the complaint. The plan of Drs. Fearne and Erskine of dashing cold water upon the surface, from the general fatality of such cases, demands a trial at the hands of every practitioner in the country. And, indeed the testimony already in its favor is such as to entitle it to general confidence.

### CHOLERA MORBUS—CHOLERA INFANTUM.

Very few cases of either of these diseases, compared with former years, have been witnessed the present season. What few cases did occur were mild and easily controlled. The state of the alimentary canal has been generally healthy, less disposed to take on disease of the above character, or in the forms of flux or diarrhœa, than formerly. Even in fevers of pretty considerable violence its integrity has been easily preserved. This state of things contrasts very strongly with the predisposition of this apparatus to disease a few years since. Then we could treat hardly anything without having it complicated with gastro-enteritis, or more or less irritation of the mucous surface of the bowels.

In his work on Animal Chemistry, Liebig occasionally throws out a speculation on etiology. He says, "in our climate hepatic diseases or those arising from excess of carbon prevail in summer; in winter pulmonary diseases or those asising from excess of oxygen are most frequent." How is the former part of this paragraph to be understood? Does the philosopher of Giessen mean to say, that hepatic diseases, and hepatic complications are more frequent in summer, because at that season of the year there is the most carbon absorbed by the skin and lungs? Or does he mean, that there is an excess taken into the system during the summer months in the food?

By the experiments of Saussure it has been clearly ascertained that the proportion of carbonic acid in the atmosphere is greater in summer than in winter, more abundant in gloomy than in bright weather, more copious in elevated situations, as on the summits of high mountains, than in the plains. These are facts in regard to the eudiometrical condition of the atmosphere. But the question as to whether there is any of this substance absorbed by the skin and lungs, or more of it in summer than in winter, remains yet to be settled.

By the entire surface of the body as well as by the lungs, oxygen, as all know, is continually being absorbed; and carbonic acid from both skin and lungs is as constantly evolved. In summer, it is very certain that there is less oxygen absorbed, and as a consequence there would be less carbonic acid evolved. This would leave the relative proportion of carbonic acid arising from the metamorphosis of the tissues in excess in the syste.n, and therefore would be equivalent to a direct absorption. On such an hypothesis there might be an excess of carbonic acid in the system during the summer. Here, however, a query might be raised to know whether in a diminished supply of oxygen the work of metamorphosis would go on so as to produce in the blood for elimination an excess, or superabundance of carbonic acid? It seems to be pretty well settled that a metamorphosis giving rise to waste in the tissues, is in a direct ratio to the amount or quantity of oxygen absorbed. In an atmosphere rich in oxygen, or where the respiratory function is increased above the healthy standard, there is always a greater waste of the tissues than where these circumstances are not present.

What now are the facts concerning the relative proportions of carbonic acid taken into the system with the food in summer and in winter? The food of the inhabitants residing in cold or Northern latitudes, as for example, in Sweden and in Kamschatka, contain from sixty to eighty per cent. of carbon, while that upon which the natives of the South prefer to feed does not contain more than twenty per cent. During the summer months, the inhabitants of temperate climates use principally fresh fruits as food; but in winter bacon and other articles rich in carbon are consumed in greatest abundance. The former contain from twelve to twenty per cent. of carbon; the latter from fifty to eighty.

According to such facts there is less carbon taken into the system in summer, the period of the greatest prevalence of our hepatic diseases, than in winter. It might be urged, however, as the activity of the respiratory process is considerably diminished during warm weather, that, therefore, the system is unable to assimilate what carbon is taken in with the food, so as to be eliminated by the skin and lungs. But then it may be replied, that the diminished condition of the respiratory function, is such as that it merely corresponds with the diminished proportion of carbon in the food.

According to the same author, the office of the liver is to separate the carbonized constituents from the blood, not for excretion, as some of the most profound physiologists of the present day suppose, but for the purpose of preparing it to re-enter the circulation, from whence it passes out of the system by the respiratory function. Whatever, therefore, interferes with the secretions of the liver exercises an influence over the proportions of carbon present in the system. Hot weather, we know, is attended with diseases of the liver; but whether these result from deficiency or an excess of the quantity of bile secreted is a question that, as far as we know, has not been decided.* If the bile, in the normal state of the system, is not excreted, as our author supposes, of course no conclusions founded on the mere retention of it in the system, as the cause of hepatic diseases, would be relevant. Again, to conclude, that the bile during summer is secreted in excess, implies that at this period the blood from which the secreted fluid is obtained, contains it in excess, an inference certainly unsupported, if as we have seen, the food during summer contains but a small proportion of carbon, and there is none at all of it, at any season of the year, that gets into the blood through either the skin or lungs.

When either the function of the skin or of the lungs is interrupted, compounds rich in carbon make their appearance in the urine, giving to that fluid a brown color. (Liebig.) Eminently both of these functions are disturbed in our autumnal fevers, and as a consequence we have the excess of carbon in the urine. Most likely the presence of this carbon in the urine is due to a deficiency of oxygen resulting from the pathological condition of the two most important inlets by which that substance finds access to the system. This being the case, the unoxydised carbon proceeding from the waste of the organs, as well as that arising from the food would necessarily seek an outlet through some of the emunctories. If this view be correct, the presence of carbon in the urine can be regarded in no other light than that of an effect of previously existing disease.

Finally, if our autumnal diseases, in which derangements of the liver are most conspicuous, arise from excess of carbon, the primary and most important indication of cure would seem to be, to administer something by which the carbon might be purged from the system. Instead of doing this

---

* It is the general opinion of physiologists, that heat increases the biliary secretion. But the presence of bile in various parts of the body, as seen in jaundice, and in our autumnal bilious diseases, results, most probably, from some mechanical obstruction in the appendages of the liver.

we use quinine, containing about *seventy-five per cent. of carbon,* and find it to be the nearest approach to a specific that is employed in the practice of medicine.

Having established it as a fact, by an experiment made on himself, that *benzoic* acid is capable of being converted, in the human system, into *hippuric* acid, Liebig says that it "places beyond all doubt the position that a mere azotised substance taken in the food can take a share, by means of its elements, in the act of transformation of the tissues, and in the formation of a secretion." "This," he continues, "throws a clear light on the mode of action of the greater number of remedies; and if the influence of caffeine on the formation of urea or uric acid should admit of being demonstrated in a similar way, we should then possess the key to the action of quinine and of the other vegetable alkalies."

Jamestown, Ohio, Oct. 10, 1845.

